# Clinical Determinants of Unsuccessful Ultrasound-Guided Hydrostatic Saline Reduction in Children with Intussusception Post-Rotavirus Vaccine Introduction: Insights from a Tertiary Care Centre in Thrissur, Kerala, India

**DOI:** 10.1007/s12098-025-05778-2

**Published:** 2025-11-03

**Authors:** Aparna Namboodiripad, Rose Xavier, Varsha Sudhir Chaudhary, Ragavi Lingam, Gibi George, Nithya Thuruthiyath, Meera Mary Alappat, Anupama Machathi, Namrata Kharat

**Affiliations:** 1 Department of Pediatrics, Jubilee Mission Medical College and Research Institute, Thrissur, Kerala, India; 2 Department of Community Medicine, Christian Medical College, Vellore, Tamil Nadu, India; 3 Department of Pediatrics, Government Medical College, Thrissur, Kerala, India; 4 School of Bioscience and Technology, Vellore Institute of Technology, Vellore, Tamil Nadu, India

**Keywords:** Laparotomy, Hydrostatic saline reduction, Pediatric intussusception, Treatment failure, Brighton Collaboration criteria

## Abstract

**Objectives:**

Intussusception (IS) is a condition in which proximal segment of the bowel invaginates into its distal segment. IS in young children can usually be managed using non-invasive methods, such as hydrostatic saline reduction (HSR), and surgery is generally reserved for cases in which non-invasive methods have failed. This study was conducted as part of a larger multicenter IS surveillance study. The objective of this analysis was to identify clinical and demographic factors associated with failed HSR in children hospitalized with IS.

**Methods:**

In this observational study, children aged < 2 y hospitalized with IS confirmed according to the Brighton Collaboration criteria at a tertiary care center in Kerala were enrolled after obtaining parental informed consent. Children who underwent spontaneous reduction were excluded. Enrolled children were followed up until discharge.

**Results:**

Of the 137 children who underwent HSR, 131 (95.6%) procedures were successful. HSR failed in six children (4.4%), and they required surgery; however, there were no deaths. The factors associated with failed HSR included low socioeconomic status [83% (5/6) vs. 32.1% (42/131); *p* = 0.042], a left-sided mass detected on ultrasonography [50% (3/6) vs. 6.9% (9/131); *p* = 0.004], and longer time from admission to the procedure (median: 5.5 vs. 3 h; *p* = 0.005).

**Conclusions:**

This study demonstrates the importance of early HSR and exercising caution when a left-sided mass is detected on ultrasonography. With timely intervention, almost all cases can be successfully managed without surgery.

## Introduction

Intussusception (IS), an invagination of the proximal segment into the distal segment of the intestine, is a common cause of intestinal obstruction in young children [1]. Some studies suggest an increased risk of IS in the first few weeks after receiving rotavirus vaccine [2]. The oral rotavirus vaccine, ROTASIIL was introduced in the Universal Immunization Program (UIP) of Kerala, India, in September 2019 [3]. Jubilee Mission Medical College and Research Institute, Thrissur in Kerala participated in a multicenter surveillance study to assess the impact of the introduction of the oral rotavirus vaccine, ROTASIIL, in the UIP on IS.

Ultrasonography is the preferred imaging modality for the diagnosis of IS owing to high specificity and sensitivity, and the lack of radiation use. Fluoroscopy-guided pneumatic enema and ultrasound-guided (USG) hydrostatic enema are the preferred management strategies [4] in the absence of contraindications, such as persistent small intestinal obstruction, cardiovascular shock, intestinal perforation, repeated failure of saline reduction, peritoneal infection, air under diaphragm, or sepsis [5]. Most cases of IS in young children do not have a pathological lead-point detected on ultrasonography, and hence, can be managed by non-surgical methods [6]. In children admitted with uncomplicated ileocolic IS, quality improvement initiatives can reduce the total cost of hospital admission and the length of stay [7]. Multiple studies having been conducted on the factors associated with successful reduction of IS in other countries [8–11], but few studies have been conducted in India. Despite the global application of hydrostatic saline reduction (HSR) in managing pediatric IS, information on its effectiveness in the Indian pediatric population, particularly in the context of socioeconomic and demographic factors, is limited. This study aimed to fill this gap and identify clinical and demographic factors associated with failed HSR in children with IS admitted to Jubilee Mission Medical College and Research Institute, Thrissur, Kerala in order to optimize clinical management protocols.

## Material and Methods

This study was conducted as part of a larger multicenter surveillance study in four states of India to assess the safety of the oral rotavirus vaccine, ROTASIIL after its introduction in the UIP. This analysis was restricted to children presenting to the Department of Pediatrics or Pediatric Casualty of a private tertiary care hospital in Thrissur, Kerala, between March 2020 and December 2022, with suspected IS. Children were eligible for enrollment if they were aged < 2 y, met the level 1 Brighton Collaboration criteria for diagnosis of IS [12], and the child’s parent or guardian provided informed consent. Further details on the study methods are provided in the introductory article [13]. Wealth distribution was divided into low, middle, and high groups using a household asset-based wealth index expressed as income tertiles. Data regarding the presence of a left-sided mass were collected from the ultrasonographic reports used to diagnose IS in each patient.

The diagnoses of all children with clinically suspected IS were confirmed using ultrasonography (USG). The primary modality of treatment of IS in the study hospital was USG HSR, performed by a radiologist, after a fasting period of ≥ 3 h. Children with contraindications to USG HSR underwent surgery, performed by a pediatric surgeon.

According to the protocol used at the study hospital, the contraindications to HSR were as follows:

1. Sickness, hemodynamic instability with evidence of sepsis (e.g., fever, leukocytosis)
2. Bowel wall showing gross edema or evidence of impending necrosis (hypoechoic areas) correlating with clinical history (e.g., onset of symptoms > 2 d prior to presentation)
3. Moderate amount of particulate ascites
4. Portal pyemia: Air in portal radicles
5. Lead point other than mesenteric lymph nodes; or
6. Lack of parental consent for HSR

Successful HSR was confirmed by observing the free gush of normal saline from the ileocecal valve. In successful cases, repeat USG was performed 24 h after the procedure to confirm the disappearance of the IS. If ultrasonography showed a residual mass obstructing the flow of saline at the end of the HSR procedure, the HSR procedure was considered to be unsuccessful.

The data were analyzed using Stata version 16.1 [14]. Participant characteristics were described using the frequency and percentage or the mean and interquartile range for categorical and continuous variables, respectively, and the statistical significance of differences between groups was assessed using chi-square tests, Fisher’s exact test, or the Wilcoxon rank-sum test, as appropriate. Two-tailed *p* values < 0.05 were considered statistically significant.

## Results

**Fig. 1:**
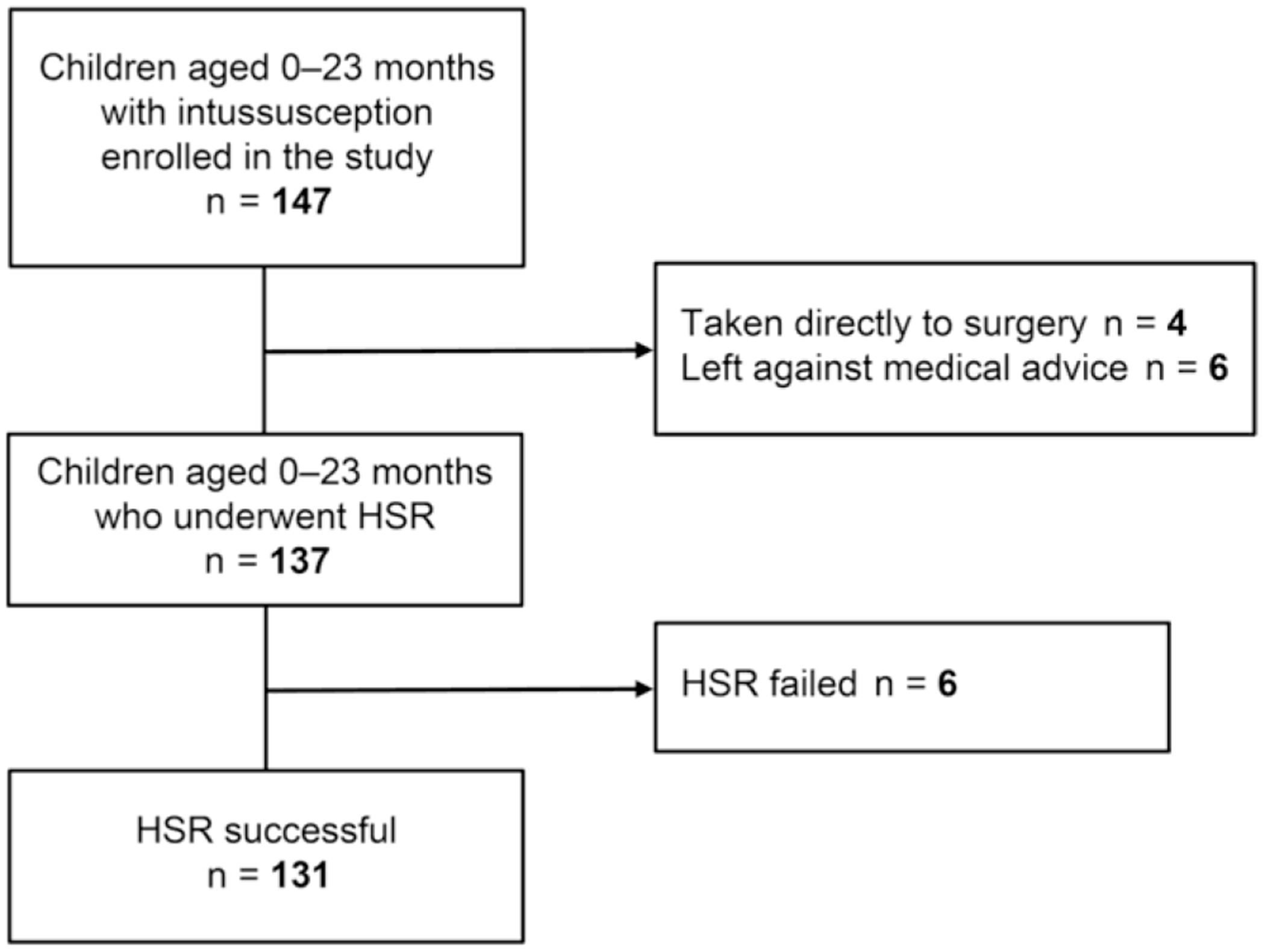
Flow chart of patient recruitment and outcomes. *HSR* Hydrostatic saline reduction

Of 147 enrolled children aged 0–23 months with IS, 137 underwent HSR, 4 were taken directly for surgery, and 6 left against medical advice and, therefore, their outcomes were unknown (Fig. 1). HSR was successful in 131 of the 137 cases and 6 reductions failed. The four children who were directly taken for surgery included two children with bowel edema detected on ultrasound with a prolonged time since onset, of whom one child also had intramural air detected in their intestine; one child with a fracture on the roof of the orbit due to a head injury; and one child with a diagnosis of Meckel’s diverticulum and suspected compromised bowel vascularity.

Of the 147 children enrolled, 90 (61.2%) were infants aged less than 12 mo, and 78 (53.1%) were male. Vomiting and abdominal pain were the most common clinical manifestations. Of the 137 children who underwent HSR, the procedure was successful in 131 (95.6%). The six children in whom HSR failed subsequently had the IS reduced under laparotomy. The sociodemographic and clinical characteristics of children with failed and successful hydrostatic saline reduction of IS are shown in Table 1. All six children with failed HSR were aged < 12 mo and had received at least one dose of oral rotavirus vaccine, whereas, of the 131 children with successful HSR, 58.8% (77/131) were aged < 12 mo and 65.6% (86/131) had received at least one dose of oral rotavirus vaccine (Table 1); however, these differences in age and vaccination status between groups was not significant (*p* = 0.081 and *p* = 0.177, respectively). Compared to children with successful HSR, children with failed HSR were significantly more likely to come from families in the lower income tertile [83% (5/6) vs. 32.1% (42/131); *p* = 0.042]; however, other characteristics (including sex, presentation with the clinical triad of vomiting, pain and blood in the stool, location of the IS, and mean length of the IS on ultrasonography) did not differ significantly between the failed and successful HSR groups (Table 1). A mass in the left hypochondrium on ultrasonography was significantly more common among children with failed HSR than in those with successful HSR [50% (3/6) vs. 6.9% (9/131); *p* = 0.004; Table 1).

The median interval from onset to admission was 1 d for both groups and did not differ significantly by group; however, the median interval from admission to the HSR procedure was significantly longer in those with failed HSR than in those with successful HSR (median: 5.5 h vs. 3 h; *p* = 0.005).

Surgical reduction was successful in five of the six children with failed HSR, and one child required surgical bowel resection. None of the six children who underwent surgery had lead points, such as Meckel’s diverticulum. No deaths occurred in either group.

## Discussion

This study showed that almost all cases of IS can be managed successfully with HSR. The only factors identified as being associated with failed HSR were lower socio-economic status, a left-sided mass, and a delay between admission and performing the procedure. In a study of 288 cases of IS conducted in China in 2014, He et al. [8] also did not identify any significant associations between age or sex and failed HSR. However, in a meta-analysis of 38 studies including 40,133 children by Kim et al. [9], and in studies by Xiaolong et al. [10] and Khorana et al. [11] conducted in China and Thailand respectively, age < 12 mo was found to be associated with an increased risk of failed HSR.

In this study, the failure rate of HSR was significantly higher in children of lower socioeconomic status. This could be attributable to the association of lower socioeconomic status with reduced access to medical care and social services, poorer-quality diet, and poor knowledge of health promotion [15]. A literature search did not identify any previous studies in India or elsewhere that have examined the socioeconomic factors affecting HSR outcomes.

Although several studies have reported an association between blood in the stool and failed HSR [8–11, 16], this was not noted in the current study. This can be attributed to the small number of children with blood in their stool providing insufficient statistical power to detect a clinically meaningful difference: Only six children presented with blood in the stool, of whom three had failed HSR. Rai et al. [17], and Wondemagegnehu et al. [18] reported that children with ileo-ileocolic IS were significantly more likely to require surgery than were those with ileocolic IS. However, in this study, the success rate of HSR was not significantly affected by the location of the IS. One possible explanation is the absence of ileo-ileocolic IS among study participants. All but two participants had ileocolic IS. Of the two participants with ileo-ileal IS, one had unsuccessful HSR.

This study found that a left-sided abdominal mass was associated with a significantly increased risk of failed HSR. This has also been reported as a risk factor for failed HSR in several previous studies [8–11].

Previous studies have shown inconsistent results regarding the effect of delays between onset and hospital admission, and delays between admission and performing the procedure on the success of HSR. In this study, the time from onset to admission did not influence the outcome of HSR, consistent with the results of studies by Tareen et al. [19] in Ireland, and Liu et al. [20] in China. However, in the systematic review by Kim et al. [9], and studies by Lehnert et al. [21] in Germany, and Fike et al. [16] in the United States, a longer interval from onset to admission was associated with a higher HSR failure rate. In studies by Lopez-Rippe et al. [22], and Williams et al. [23], both conducted in the United States, the failure rate was not associated with the interval from diagnosis of IS to attempting HSR. In contrast, Lampl et al. [24], in another study conducted in the United States, found that the HSR failure rate was higher if the procedure was delayed, as in the present study.

The HSR success rate of 95.6%, with no deaths, in this study is higher than that reported in previous studies conducted in India and elsewhere. A previous study conducted in Kerala in 2016 in the same institution as the present study, [25] reported 80.8% success rate, and Fahiem-Ul-Hassan et al. [26] reported a 92.1% success rate in a study conducted in Jammu and Kashmir, India. Sacks et al. [27] reported an 86% success rate in a study conducted in Israel, and Yadav et al. reported a 92.85% success rate in a study conducted in Nepal [28]. This study was conducted in Kerala, which has a development index, including indicators such as literacy and access to health care, comparable to that of high-income countries [29]. The participating center has round-the-clock diagnostic ultrasound facilities and staff trained in IS reduction, which may have further contributed to successful HSR. Moreover, as the study site was a private institution, the patient population may reflect a higher socioeconomic profile than that seen in government hospitals. These factors probably contributed to the high success rate at authors’ institution. Hence, the findings of this study may not be generalized to clinical practice in other settings, especially to limited-resource settings and populations with different socioeconomic, geographic, and demographic characteristics.

A strength of this study is the use of standard case definition and data-collection procedures. The main limitation of this study is the limited statistical power to detect factors associated with HSR failure owing to the high success rate of HSR in authors’ center, with only six cases of HSR failure. To better identify factors associated with HSR failure and enhance the generalizability of the findings to varied clinical contexts, larger multi-center studies in diverse regions of India, encompassing populations with a broader range of socioeconomic status, are warranted.

## Conclusions

This is one of the few studies from India on factors associated with HSR failure. Therefore, it adds to the understanding of the factors associated with HSR failure in India. It identified low socioeconomic status, a left-sided mass on ultrasonography, and a longer time from admission to the procedure as factors associated with HSR failure. The practical implication of this study for pediatric healthcare providers is that it demonstrates the importance of early intervention for IS, particularly in resource-limited settings where delays between admission and the procedure are more likely. Clinicians should carefully evaluate the ultrasonographic findings and exercise caution when a left sided mass is observed. The socioeconomic disparities between children with successful and failed HSR highlight the importance of equitable access to timely diagnostics and trained staff, and the need for systemic support in making healthcare accessible to families with low socioeconomic status. This study demonstrates the importance of early HSR and exercising caution when a left-sided mass is detected on ultrasonography and shows that with timely intervention, almost all cases of IS can be successfully managed by HSR, without surgery. However, larger multicenter studies are needed to identify factors associated with HSR failure in India.

## Supplementary Material

Raw Data

## Figures and Tables

**Table 1:** Comparison of the sociodemographic and clinical characteristics of children with failed and successful hydrostatic saline reduction of intussusception

Variable	Failed HSR *N* = 6	Successful HSR *N* = 131	*p*
Age group			0.081**
< 12 mo	6 (100)	77 (58.8)	
12–23 mo	0 (0)	54 (41.2)	
Sex			0.682**
Male	4 (67)	67 (51.1)	
Female	2 (33)	64 (48.9)	
Socioeconomic status (tertile)			0.042
Lower	5 (83)	42 (32.1)	
Middle or upper	1 (17)	89 (67.9)	
Rotavirus vaccination status			0.177**
Unvaccinated	0 (0)	45 (34.4)	
Vaccinated with at least one dose	6 (100)	86 (65.6)	
Triad of vomiting, crying (pain) and blood in the stool			0.140**
Yes	3 (50)	29 (22.1)	
No	3 (50)	102 (77.9)	
Location of Intussusception			0.086**
Ileocolic	5 (83.3)	130 (99.2)	
Ileoileal	1 (16.7)	1 (0.8)	
Length of mass (cm), median (IQR)	4.6 (3.8–8.6)	4 (3.4–5.5)	0.403^#^
Left-sided mass			0.004*
Yes	3 (50)	9 (6.9)	
No	3 (50)	122 (93.1)	
Time from onset to admission (d), median (IQR)	1 (0–1)	1 (0–2)	0.183#
Time from admission to procedure (h), median (IQR)	5.5 (4–12)	3 (2–4)	0.005^#^

*HSR* Hydrostatic saline reduction; *IQR* Interquartile range; *IS* Intussusception; *US* Ultrasonography

The values are given as n (%), unless otherwise specified

** Fisher’s exact test

# Wilcoxon rank-sum test

* Chi-square test with Yates correction
